# Hepatic arterial infusion chemotherapy with gemcitabine and 5-fluorouracil or oral S-1 improves the prognosis of patients with postoperative liver metastases from pancreatic cancer

**DOI:** 10.3892/mco.2013.152

**Published:** 2013-07-23

**Authors:** HIDEHIRO TAJIMA, HIROHISA KITAGAWA, TOMOYA TSUKADA, KOICHI OKAMOTO, SHIN-ICHI NAKANUMA, SEISHO SAKAI, ISAMU MAKINO, HIROYUKI FURUKAWA, HIRONORI HAYASHI, KATSUNOBU OYAMA, MASAFUMI INOKUCHI, HISATOSHI NAKAGAWARA, TOMOHARU MIYASHITA, HIROSHI ITOH, HIDETO FUJITA, HIROYUKI TAKAMURA, ITASU NINOMIYA, SACHIO FUSHIDA, TAKASHI FUJIMURA, TETSUO OHTA, WATARU KODA, TETSUYA MINAMI, YASUJI RYU, JUNICHIRO SANADA, TOSHIFUMI GABATA, OSAMU MATSUI, YOSHIMICHI SAI

**Affiliations:** 1 Department of Gastroenterological Surgery and, Division of Cancer Medicine, Graduate School of Medical Science, Kanazawa University, Kanazawa, Ishikawa 920-8641, Japan; 2 Department of Radiology, Division of Cancer Medicine, Graduate School of Medical Science, Kanazawa University, Kanazawa, Ishikawa 920-8641, Japan; 3 Division of Pharmacy, Kanazawa University Hospital, Kanazawa, Ishikawa 920-8641, Japan

**Keywords:** pancreatic cancer, liver metastasis, hepatic arterial infusion, gemcitabine, 5-fluorouracil, S-1

## Abstract

Hepatic metastasis is a common cause of treatment failure following resection of pancreatic cancer. In this study, we report our results of hepatic arterial infusion (HAI) chemotherapy with gemcitabine (GEM) plus 5-fluorouracil (5-FU) or oral S-1 treatment for postoperative liver metastases from pancreatic cancer. Seven patients with postoperative liver metastases from pancreatic cancer received HAI with GEM plus 5-FU or oral S-1 between October, 2008 and September, 2010 at Kanazawa University Hospital (Kanazawa, Japan). Three out of the 7 cases exhibited a partial response (PR) according to Response Evaluation Criteria in Solid Tumors (RECIST) and stable disease (SD) was achieved in 3 out of the 7 cases (response rate, 85.7%). A decrease in serum tumor marker CA 19-9 levels was observed after 10 HAI treatment cycles in 5 out of the 7 cases. The median time to treatment failure was 8 months (range, 0–17 months). Adverse events included grade 3 leukocytopenia in 1 case and anemia in all 7 cases, although 5 out of the 7 patients were anemic prior to HAI therapy. Grade 2 thrombocytopenia was also observed in 2 cases. Non-hematological events, such as nausea, diarrhea, liver injury or neuropathy and life-threatening toxicities were not reported; however, 6 patients (85.7%) developed catheter-related complications and the HAI catheter and subcutaneous implantable port system had to be removed. These findings demonstrated that HAI may deliver high doses of chemotherapeutic agents directly into the tumor vessels, producing increased regional levels with greater efficacy and a lower incidence/severity of systemic side effects. In conclusion, HAI chemotherapy is a safe and effective treatment for liver metastases from pancreatic cancer.

## Introduction

Pancreatic cancer is one of the major causes of cancer-related mortality worldwide, with a 5-year survival rate of <5% (1,2). For patients with localized disease, radical surgery may provide long-term benefits. However, even in patients who undergo resection, the reported 5-year survival rate remains low (7–24%) and the median survival is only ~1 year in most patient series, indicating that surgery alone is generally inadequate. Even following curative resection, patients with pancreatic cancer are likely to experience a 50–80% local recurrence rate and a 25–50% risk of developing distant metastases (3). Adjuvant chemotherapy with gemcitabine (GEM), the key drug used in the treatment of pancreatic cancer, improves the survival of patients with resectable pancreatic adenocarcinoma compared to resection alone (4), although to a limited extent.

However, 20–30% of patients are unable to receive the designated therapy due to postoperative complications, delayed surgical recovery and/or early disease recurrence (5,6). To improve the therapeutic results of resected pancreatic cancer, it is critical to optimize the postoperative management of liver metastases, which frequently constitute the major determining factor of prognosis.

An alternative treatment option that may be beneficial in pancreatic cancer patients with liver metastases is the hepatic arterial infusion (HAI) of chemotherapeutic agents. This treatment option has been applied to patients with primary or metastatic hepatic malignancies that are confined to the liver and is soundly based on physiological and pharmacological factors. First, liver metastases that grow >2–3 mm depend on the hepatic artery for vascularization, whereas normal liver tissues are perfused by the portal vein (7,8). Second, HAI therapy allows drug delivery to hepatic metastases not achievable by systemic administration, particularly of drugs with a high systemic clearance rate (9). Third, first-pass hepatic extraction of certain drugs results in lower systemic concentrations and, thus, few systemic toxicities (10). Phase I studies of HAI chemotherapy with GEM in patients with liver malignancies have been previously published (8–10). Moreover, results from our recent pilot study suggest that HAI chemotherapy with GEM and 5-fluorouracil (5-FU) is safe and beneficial for the treatment of postoperative metastatic tumors confined to the liver, even in patients with poor general condition (11).

Over the past few years, we have expanded the number of cases treated with HAI chemotherapy with GEM at our institution to include cases with other metastases in addition to liver metastases, by the addition of oral S-1 in lieu of 5-FU. S-1 is an oral fluorinated pyrimidine compound developed by Taiho Pharmaceutical Co., Ltd., (Tokyo, Japan). The administration of oral S-1 is more convenient and simulates the effect of continuous infusion of 5-FU. The safety and effectiveness of the combination chemotherapy with GEM and S-1 for advanced pancreatic cancer were reported by previous studies (12–14) and a phase III (GEST) trial in Japanese patients demonstrated that S-1 was not inferior to GEM (15). In this study, we present the final results of the patients who were treated with HAI with GEM plus 5-FU or HAI with GEM and oral S-1.

## Materials and methods

### Patient eligibility

Seven patients with postoperative liver metastases from pancreatic cancer underwent HAI with GEM between October, 2008 and September, 2010 at Kanazawa University Hospital (Kanazawa, Japan). Patients with metastases confined to the liver following curative (R0) resection of the pancreatic primary adenocarcinoma underwent HAI with GEM plus 5-FU (GEM+5-FU group). However, patients with metastases confined to the liver following non-curative (R1 or R2) resection or cases that involved metastases to other organs along with liver metastases that may dictate prognosis, underwent HAI with GEM and oral S-1 administration (GEM+S-1 group). Written informed consent was obtained from each patient prior to enrollment in the study and the treatment was undertaken with the approval of the local Medical Ethics Committee.

The baseline characteristics of the patients are listed in Table I. Five out of the 7 patients received GEM plus 5-FU treatment and 2 received GEM plus S-1 treatment. The male:female ratio was 5:2. The median patient age was 64.9 years (range, 60–71 years). The Eastern Cooperative Oncology Group performance status score was 0 in all patients in this study. Four patients had received preoperative chemotherapy with GEM and oral S-1 and adjuvant chemotherapy with GEM had been administered to 5 out of the 7 patients prior to the appearance of liver metastases. The interval between surgery and the appearance of liver metastases was 7 months (range, 3–11 months). The median standard liver volume [SLV (ml) = 706.2 × body surface area (BSA) + 2.4] was 1.1 l (range, 9.0–1.3 l) (16).

### Catheter placement and treatment regimen

**Figure 1:**
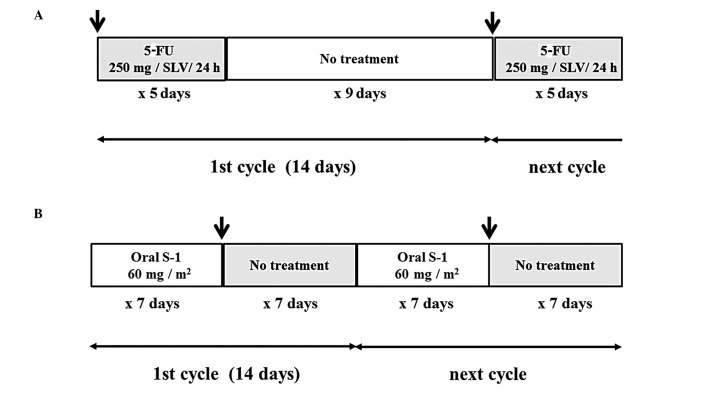
Treatment regimens of (A) gemcitabine (GEM) + 5-fluorouracil (5-FU) and (B) GEM+S-1 groups. (A) In the GEM+5-FU group, an 800-mg/SLV dose of GEM was administered over 30 min (arrow). Following GEM infusion, a 250-mg/SLV dose of 5-FU was administered continuously over 24 h on days 1–5, comprising 1 cycle of therapy. Each treatment cycle was continued biweekly on hospital days 1–6. (B) In the GEM+S-1 group, 60 mg/m^2^/day S-1 was administered for 7 consecutive days and an 800-mg/SLV dose of GEM was administered on day 8 (arrow). Each treatment cycle was continued biweekly in the outpatient clinic.

An intrahepatic arterial catheter was percutaneously implanted following hepatic arteriography via a right femoral puncture to deliver chemotherapy. The catheter tip was placed in the hepatic artery proper by a radiologist. The catheter was then connected to a subcutaneous implantable port system, located in the lower right abdominal area. In the GEM+5-FU group, an 800-mg/SLV dose of GEM was dissolved in 50 ml of saline for administration over a 30-min period using a bedside pump. Following GEM infusion, a 250-mg/SLV dose of 5-FU dissolved in 50 ml of saline was infused continuously over 24 h on days 1–5, comprising 1 cycle of therapy. In case 1, only 400 mg of GEM was administered, due to the development of leukocytopenia (17). Each treatment cycle was continued biweekly on hospital days 1–6 (Fig. 1A). In the GEM+S-1 group, 60 mg/m^2^/day of S-1 was administered for 7 consecutive days and an 800-mg/SLV dose of GEM was administered on day 8 as in the GEM+5-FU group. Each treatment cycle was continued biweekly in the outpatient clinic (Fig. 1B).

### Assessment of response

Response to treatment was determined based on the following measures: results of physical examination, complete blood counts, biochemical tests and chest and abdominal radiography were obtained prior to the initiation of each cycle. Serum CA 19-9 was measured monthly and changes in this tumor marker were assessed prior to and following 10 HAI cycles. Follow-up contrast-enhanced computed tomography was performed upon completion of every 5 cycles, or more frequently for cases showing clinical deterioration. The response rate was evaluated in accordance with the Response Evaluation Criteria in Solid Tumors (RECIST) (18). A complete response (CR) was defined as the disappearance of all evidence of disease and normalization of tumor markers persisting for at least 2 weeks. A partial response (PR) was defined as a >30% reduction on uni-dimensional tumor measurements, without the appearance of any new lesions or progression in any existing lesion. Progressive disease (PD) was defined as any of the following: i) a 20% increase in the sum of the products of all measurable lesions; ii) the appearance of any new lesion; or iii) the reappearance of any lesion that had previously disappeared. Stable disease (SD) was defined as a tumor response that did not fulfill the criteria for CR, PR or PD.

In the GEM+5-FU group, HAI of 5-FU was terminated after 10 cycles and administration of oral S-1 was initiated. Patients in the two groups received GEM HAI and administration of oral S-1 in the outpatient clinic for as long as possible, i.e., for as long as they exhibited no tumor regrowth or the appearance of any new lesions and were free of HAI catheter-related problems. The median survival time (MST) was calculated from the initiation of the study treatment until death and determined according to the Kaplan-Meier method.

## Results

In 6 out of the 7 cases, >10 cycles of HAI chemotherapy were administered. In a single case (case 5), the HAI catheter and subcutaneous implantable port system had to be removed after eight cycles due to a problem with the tube. Based on RECIST, PR was achieved in 2 out of the 7 cases and SD was achieved in 4 (response rate, 85.7%). CR was not achieved in any of the cases, whereas PD was observed in 1 case. In 5 out of the 7 cases, decreases in the serum tumor marker CA 19-9 levels were observed after 10 cycles of HAI treatment. The median time to treatment failure was 8 months (range, 0–17 months). The initial disease progression factor was nodal and lung metastasis in 3 cases and local recurrence plus peritoneal dissemination in 2. The overall survival time from the initiation of the study treatment until death was 17.4 months (range, 11–26 months) (Table II).

**Figure 2:**
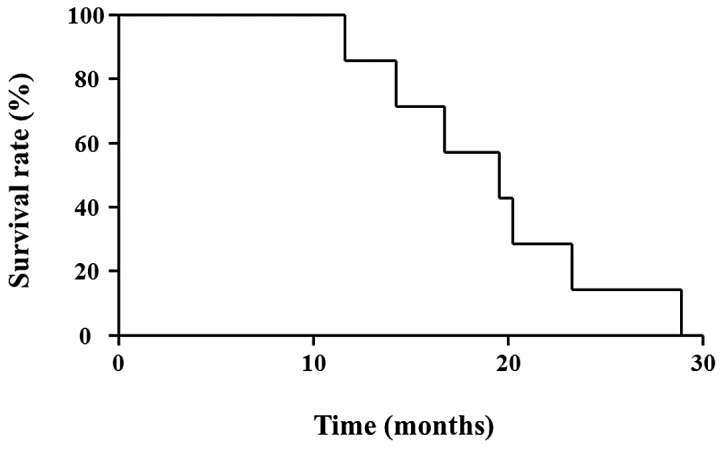
Overall survival curve for patients from the initiation of the hepatic arterial infusion (HAI) study treatment. All 7 patients eventually succumbed to the primary disease. The median survival time (MST) was 22.4 months.

Adverse events are listed in Table III. Grade 3 leukocytopenia was observed in case 1; this patient was not able to receive adjuvant systemic chemotherapy due to grade 2 leukocytopenia prior to HAI. Leukocytopenia was also observed in 1 of the remaining 6 cases. The patients were anemic; however, 5 out of the 7 patients had developed anemia prior to HAI therapy. Grade 2 thrombocytopenia was observed in 2 cases. Non-hematological events, such as nausea, diarrhea, liver injury (AST/ALT increase), or neuropathy were not observed. Of note, there were no life-threatening toxicities. However, catheter-related complications (arterial thrombosis or catheter dislocation) occurred in 6 cases (85.7%) and the HAI catheter and subcutaneous implantable port system had to be removed (Table II). All 7 patients eventually succumbed to the primary disease. The MST was 22.4 months (Fig. 2).

## Discussion

Pancreatic cancer is almost always fatal, with a 5-year survival rate of <5% (1,2). Surgery remains the only curative option and usually consists of radical pancreatic resection, including wide lymph node dissection and complete removal of the extra-pancreatic nerve plexus of the superior mesenteric artery or celiac axis (19,20). Adjuvant chemotherapy improves the survival of patients with resectable pancreatic adenocarcinoma compared to resection alone (4), although to a limited extent. However, 20–30% of patients are unable to receive the designated therapy due to postoperative complications, delayed surgical recovery and/or early disease recurrence (5,6). In particular, the appearance of liver metastases early in the postoperative period significantly contributes to a poor prognosis in postoperative patients. For these patients, HAI chemotherapy, which has less of an effect on the body as a whole, may provide an effective treatment alternative to standard adjuvant chemotherapy.

Arterial infusion chemotherapy with GEM and 5-FU has been reported as a treatment for locally advanced pancreatic cancer and liver metastases from pancreatic cancer (10,21,22). Furthermore, in previous phase I studies, HAI chemotherapy with GEM was well-tolerated up to 1,000 mg/m^2^ infused over 400 min (8,9).

According to the pharmacokinetics of GEM, when 1,000 mg/m^2^ of GEM is administered via intravenous infusion over 30 min, the average maximum plasma concentrations reach 21,865±4,165 ng/ml by 15 min. The flow volume of the hepatic artery proper is reportedly ~330 ml/min (11). When an 800-mg dose of GEM is infused into the hepatic artery proper over a 30-min period, the local plasma concentration in the liver reaches ~80,000 ng/ml by 30 min. Vogl *et al*(8) reported that the maximum tolerated dose of HAI chemotherapy with GEM was 1,400 mg/m^2^. Conversely, the plasma concentration of 5-FU with a 250-mg infusion into the hepatic artery proper over a 24-h period was 0.5 μg/ml. This concentration is equal to that obtained following administration of 30 mg/kg (1,350 mg in the reported patient) of 5-FU over a 24-h period (23). In addition, Maruyama *et al*(24) reported that when 1,000–1,500 mg of 5-FU was infused into the hepatic artery over a period of 5 h, the maximum plasma concentration was 0.48 μg/ml on average, without the development of any grade 3 adverse effects. Super-selective HAI may deliver high doses of chemotherapeutic agents into the tumor vessels, producing increased regional levels with higher effectiveness and lower incidence/severity of systemic side effects. In this study, the response rate was 85.7%, despite 6 out of the 7 cases having received systemic chemotherapy with GEM prior to HAI. Moreover, no severe toxicity developed with this therapy. These findings indicate that HAI chemotherapy is safe and effective for the treatment of postoperative liver metastasis from pancreatic carcinoma. The drawbacks of HAI chemotherapy include problems with the catheter and the appearance of new lesions outside the liver. In this study, 6 out of the 7 cases eventually required removal of the HAI catheter and the subcutaneous implantable port system due to problems with the tube, and new lesions outside the liver appeared in all 7 patients. Even in the GEM+5-FU group, we performed HAI with GEM plus oral S-1 therapy after 10 cycles under the hypothesis of the appearance of extra-hepatic metastases. Moreover, patients who did not undergo R0 surgery for primary lesions and patients suspected of having extra-hepatic metastases were assigned to the GEM+S-1 group.

Although the safety of GEM plus S-1 therapy was previously demonstrated in the GEST trial (15), the occurrence of adverse events was greater in the phase I study of neoadjuvant chemotherapy (NAC) for resectable pancreatic cancer performed in our department. In addition, NAC with GEM plus S-1 was not well-tolerated (25). Nakahira *et al*(26) reported that pretreatment with S-1 enhances the GEM effects on pancreatic cancer xenografts. The mechanism underlying these enhanced effects is considered to be 5-FU-induced upregulation of human equilibrative nucleoside transporter 1, the major mediator of GEM cellular uptake. In our trial, S-1 was administered for 14 consecutive days prior to GEM, which may explain the greater number of adverse events observed in our study. However, to maximize the effect of GEM, it is recommended that S-1 be administered prior to GEM. By using a combination of oral S-1 and HAI of GEM, effective amounts of the two chemotherapeutic agents were reached in the liver and the systemic side effects were reduced.

In conclusion, HAI chemotherapy is safe and effective for the treatment for postoperative metastases from pancreatic cancer confined to the liver. A clinical phase I trial of HAI chemotherapy with GEM plus 5-FU or oral S-1 is currently being undertaken, which includes the measurement of GEM concentration in the peripheral blood of patients in order to determine the optimal dose.

## Figures and Tables

**Table I:** Patient characteristics.

Case number	1	2	3	4	5	6	7
Age (years)	61	62	69	71	60	66	65
Gender	F	M	M	M	F	M	M
Performance status	0	0	0	0	0	0	1
Tumor location	H	H	BT	BT	BT	H	BT
Residual tumor	0	0	0	0	0	0	1
Preoperative chemotherapy	+	+	−	−	−	+	+
Postoperative chemotherapy	−	+	−	+	+	+	+
Interval between operation and liver metastases (months)	5	10	3	10	5	11	5
Body surface area (m^2^)	1.6	1.7	1.8	1.5	1.3	1.7	1.5
Standard liver volume (l)	1.1	1.2	1.3	1.1	0.9	1.2	1.1
Other metastatic lesion prior to HAI	−	−	−	−	−	P	−
Group	GEM+5-FU	GEM+5-FU	GEM+5-FU	GEM+5-FU	GEM+5-FU	GEM+S-1	GEM+S-1

F, female; M, male; GEM, gemcitabine; 5-FU, 5-fluorouracil; H, head; BT, body and tail; P, peritoneal dissemination; HAI, hepatic arterial infusion.

**Table II:** Treatments and responses.

Case number	1	2	3	4	5	6	7
GEM administration (cycles)	13	40	23	10	8	15	12
5-FU administration (cycles)	10	10	10	10	6	0	0
S-1 administration (cycles)	3	30	13	0	2	15	12
Response	SD	PR	PR	PR	SD	SD	PD
TTF (months)	15	17	7	8	3	6	0
Other metastatic lesion	L, N	N, P	Lg	L, Lg	N	P	Lg
Other chemotherapy	Tx	Tx	Tx	Tx	Tx	Tx	Tx
Other therapy	RT	RT	−	−	−	−	−
Survival following HAI (months)	23	26	13	20	13	16	11
Catheter problems	+	+	−	+	+	+	+
CA19-9 prior to HAI (U/ml)	138	14	311	2,073	43,460	423	37
CA19-9 following 10 HAI cycles (U/ml)	33	65	221	811	32,200	34	1,060

GEM, gemcitabine; 5-FU, 5-fluorouracil; SD, stable disease; PR, partial response; PD, peritoneal dissemination; TTF, time to treatment failure; L, local recurrence; N, lymph node metastasis; Lg, lung metastasis; Tx, taxane; RT, radiation therapy; HAI, hepatic arterial infusion; CA 19-9, carbohydrate antigen 19-9.

**Table III:** Treatment toxicities (NCI-CTC grade)

Case number	1	2	3	4	5	6	7
Anemia	2^a^	2^a^	1	1^a^	2^a^	2^a^	1
Leukocytopenia	3^b^	0	0	0	0	2^a^	0
Thrombocytopenia	2^a^	1	0	2	0	1^a^	0
Nausea	0	0	0	0	0	0	0
Diarrhea	0	0	0	0	0	0	0
Liver injury	0	0	0	0	0	0	0
Neuropathy	0	0	0	0	0	0	0

a Grade 1 prior to hepatic arterial infusion (HAI) initiation,

b grade 2 prior to HAI initiation.

NCI-CTC, National Cancer Institute-Common Toxicity Criteria.
